# Unravelling the Unseen: A Case Series Exploring the Enigmas of Traumatic Optic Neuropathy

**DOI:** 10.7759/cureus.75546

**Published:** 2024-12-11

**Authors:** Soham N Naik, Dhruvil V Nayak

**Affiliations:** 1 Department of Radiodiagnosis, Sir Takhtasinhji General Hospital, Bhavnagar, IND; 2 Department of Ophthalmology, Sir Takhtasinhji General Hospital, Bhavnagar, IND

**Keywords:** neuro-ophthalmology, neuro-radiology, optic nerve injury, orbital trauma, traumatic vision loss

## Abstract

Traumatic optic neuropathy (TON) is a rare condition resulting from damage to the optic nerve due to craniofacial trauma. It can present as direct or indirect injuries, with mechanisms ranging from mechanical disruption by fractures in direct TON to transmitted forces causing shearing and ischemia in indirect TON. These injuries often lead to significant visual impairment or complete vision loss, requiring timely diagnosis and intervention. This case series explores five distinct presentations of TON and its related syndromes: direct TON caused by a displaced optic canal fracture, managed with surgical decompression and corticosteroids; indirect TON associated with orbital fractures, treated with high-dose corticosteroids; a complete optic nerve tear, where surgical intervention was deferred due to irreparable damage; TON complicated by cavernous sinus thrombosis, requiring anticoagulation, antibiotics, and corticosteroids; and traumatic chiasmal syndrome presenting with bitemporal hemianopia, treated with corticosteroids. Advanced imaging modalities, particularly computed tomography (CT) and magnetic resonance imaging (MRI), played a pivotal role in diagnosing the extent and mechanism of injuries, guiding individualized management strategies. Outcomes varied, with partial visual recovery achieved in direct and indirect TON, while cases involving severe structural disruption, such as optic nerve tears and chiasmal injuries, showed minimal or no improvement. The TON case with cavernous sinus thrombosis demonstrated thrombosis resolution but without visual restoration. This series emphasizes the importance of a multidisciplinary and tailored approach, combining advanced imaging and medical or surgical therapies, to optimize outcomes in TON and its variants. However, the limitations of current therapies in cases of severe nerve disruption highlight the critical need for advancements in neuroprotective and regenerative treatments to address this challenging condition.

## Introduction

The optic nerve comprises axons of retinal ganglion cells (RGCs) and support cells. At 50 mm in length, it consists of the following four segments: intraocular (1 mm), intraorbital (24 mm), intracanalicular (9 mm), and intracranial (16 mm). The ON may be injured in trauma, resulting in visual loss, and this is known as traumatic optic neuropathy (TON). TON occurs from either direct or indirect trauma [1].

In cases of direct trauma, stress is exerted directly onto the ON, often from orbital fracture fragments or mechanical impact. Conversely, indirect trauma is more common, where force is transmitted through the surrounding facial tissues and bones. This indirect impact, often at the junction between mobile and fixed nerve segments (such as the intraorbital and intracanalicular sections), can compress and damage the pial vessels within the canal, limiting blood flow to the ON [2]. In a study of 42 patients with TON, the frequency of site of injury was intracanalicular (71.4%) > orbital apex (16.7%) > both (11.9%) [3].

In patients with a rapid post-traumatic decrease in visual acuity, high-resolution CT of the orbital apex should be performed to evaluate for possible fracture and to guide surgical intervention. If there are no contraindications, MRI imaging should be performed to look for signal intensity changes in the optic nerve [4].

Computed tomography (CT) is considered the top choice for evaluating orbital trauma. The best protocol is to obtain thin-section axial CT scans and perform multiplanar reformation. Axial and coronal T1-weighted images without fat suppression, axial and coronal short tau inversion recovery (STIR) images, axial diffusion-weighted images (DWI), and axial and coronal post-contrast fat-suppressed T1-weighted images are common clinical MRI protocols for the optic nerve. MRI magnetic field strength of 1.5 T or 3 T is recommended, and the slice thickness should be less than 3 mm. Both axial and coronal images should include the orbital apex and cavernous sinus [4,5].

This article delves into a series of notable cases illustrating intriguing findings in post-traumatic optic nerve injuries. By shedding light on these cases, we aim to enrich the clinical and radiologic understanding of TON's diagnostic nuances, prognostic factors, and therapeutic dilemmas. Our case series underscores the heterogeneity of TON, highlighting the pivotal role of individualized management tailored to the distinct clinical scenario.

## Case presentation

Case 1

A 32-year-old male presented with acute vision loss in the right eye following blunt craniofacial trauma. Clinical examination revealed no light perception in the right eye, while vision in the left was 6/6 unaided. A significant afferent pupillary defect was observed on the right side; the colour vision was impaired on Ishihara visual charts. External examination demonstrated right-sided periorbital oedema and ecchymosis, indicative of facial trauma. Neurological evaluation was unremarkable, with intact motor and sensory functions and no deficits in cranial nerves other than the optic neuropathy. The anterior segment showed subconjunctival haemorrhage and intraocular pressure was 18 mm hg, measured by a non-contact tonometer. Ophthalmological findings confirmed the diagnosis of TON.

**Figure 1:**
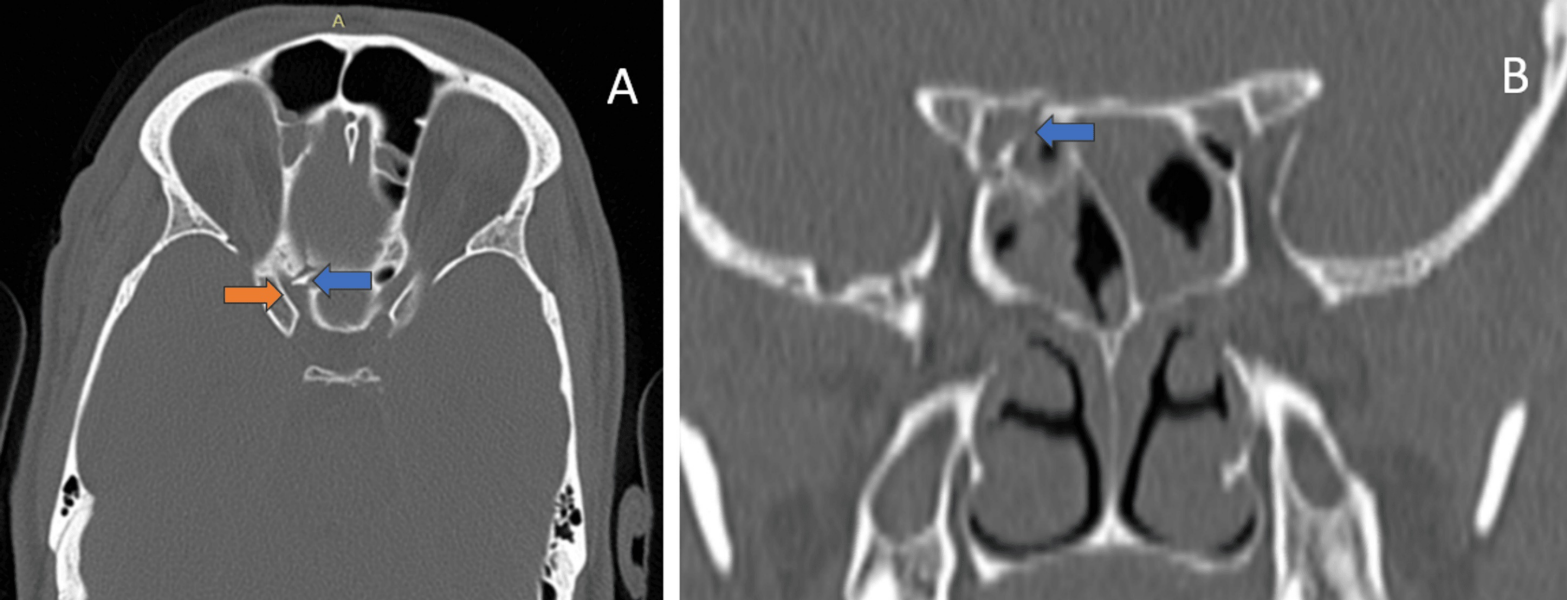
(A) Axial non-contrast computed tomography image of the orbit shows a sharp linear fracture fragment measuring 5 mm (blue arrow) impinging the right optic nerve canal (orange arrow). (B) Coronal non-contrast computed tomography image of the orbit shows a fracture of the medial wall and roof of right side optic nerve canal (blue arrow).

CT scans revealed a displaced fracture of the medial wall of the right optic canal, with a 5 mm bone fragment impinging on the right optic nerve, as shown in Figures 1A-1B. Additional fractures included mildly displaced fractures of the right orbital floor, medial wall, and lesser wing of the sphenoid, as well as comminuted fractures of the bilateral lateral and right medial pterygoid plates.

**Figure 2:**
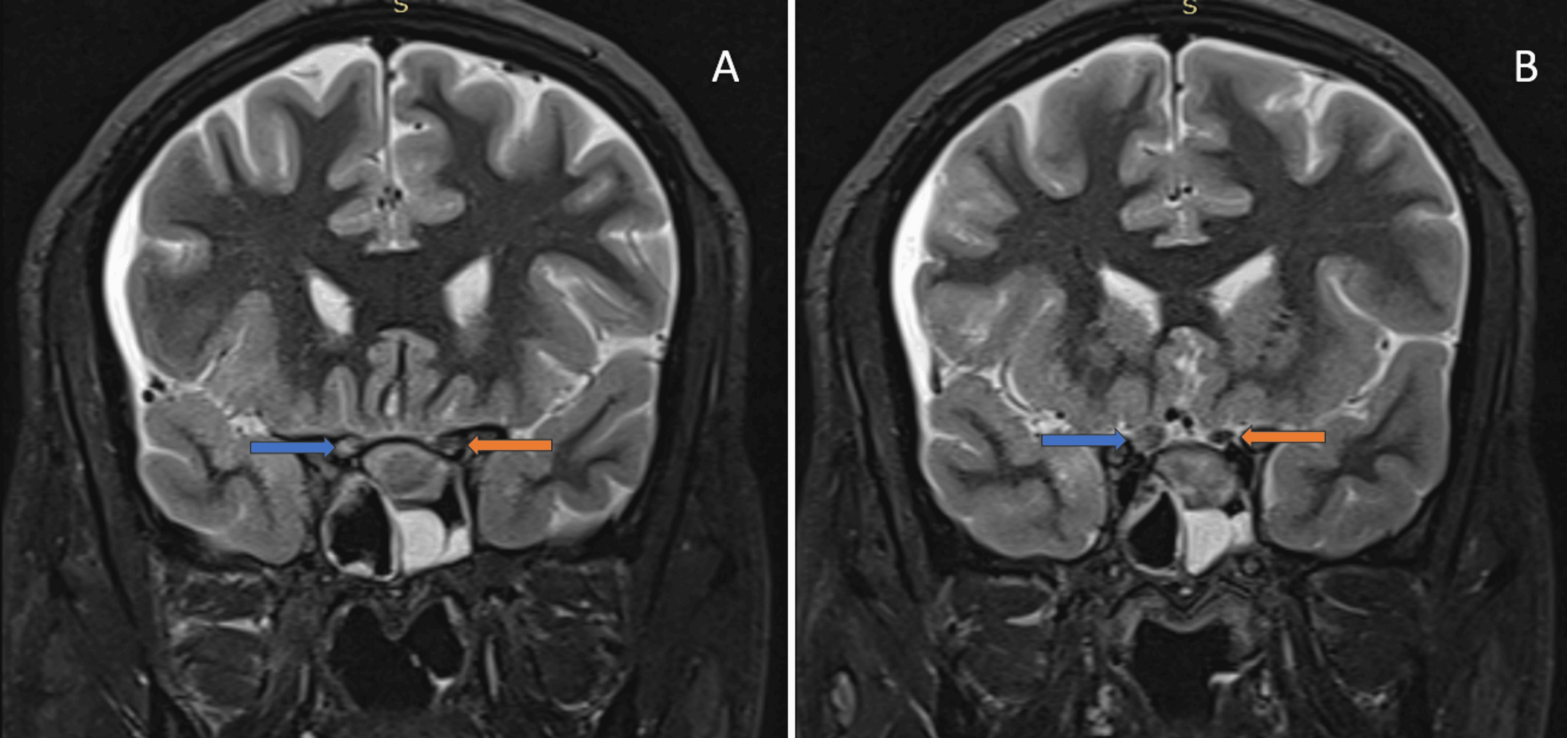
(A) Coronal MRI short tau inversion recovery (STIR) image shows markedly increased signal intensity in the intracanalicular segment of the right optic nerve (blue arrow) compared to the normal signal intensity of the intracanalicular segment of the left optic nerve (orange arrow). (B) Coronal MRI STIR image shows swelling with markedly increased signal intensity in the intracranial segment of the right optic nerve (blue arrow) compared to the normal signal intensity of the intracranial segment of the left optic nerve (orange arrow).

MRI demonstrated swelling and hyperintensity of the intracanalicular and intracranial segments of the right optic nerve on STIR sequences, extending to the optic chiasm, as shown in Figures 2A-2B. These imaging features suggested the diagnosis of direct TON.

The patient underwent endoscopic optic nerve decompression (EOND) surgery to alleviate the mechanical compression caused by the displaced fracture fragment. Postoperatively, high-dose intravenous methylprednisolone was administered at 1 gram per day, divided into four doses, for five days. This was followed by a transition to oral prednisolone at a dose of 1 mg/kg per day, maintained for three days, and tapered over the subsequent nine days. The dual approach of surgical decompression and pharmacological therapy aimed to minimize inflammation and promote optic nerve recovery.

The postoperative evaluation revealed partial improvement in visual perception in the right eye, best corrected visual acuity improved to 6/60 on the Snellen visual acuity chart; however, colour vision remained defective on the Ishihara visual chart. The patient exhibited no further visual deterioration or new complications during recovery. Continued follow-up for one month highlighted the success of the combined surgical and medical approach, with the patient showing a favourable trajectory towards visual recovery.

Case 2

**Figure 3:**
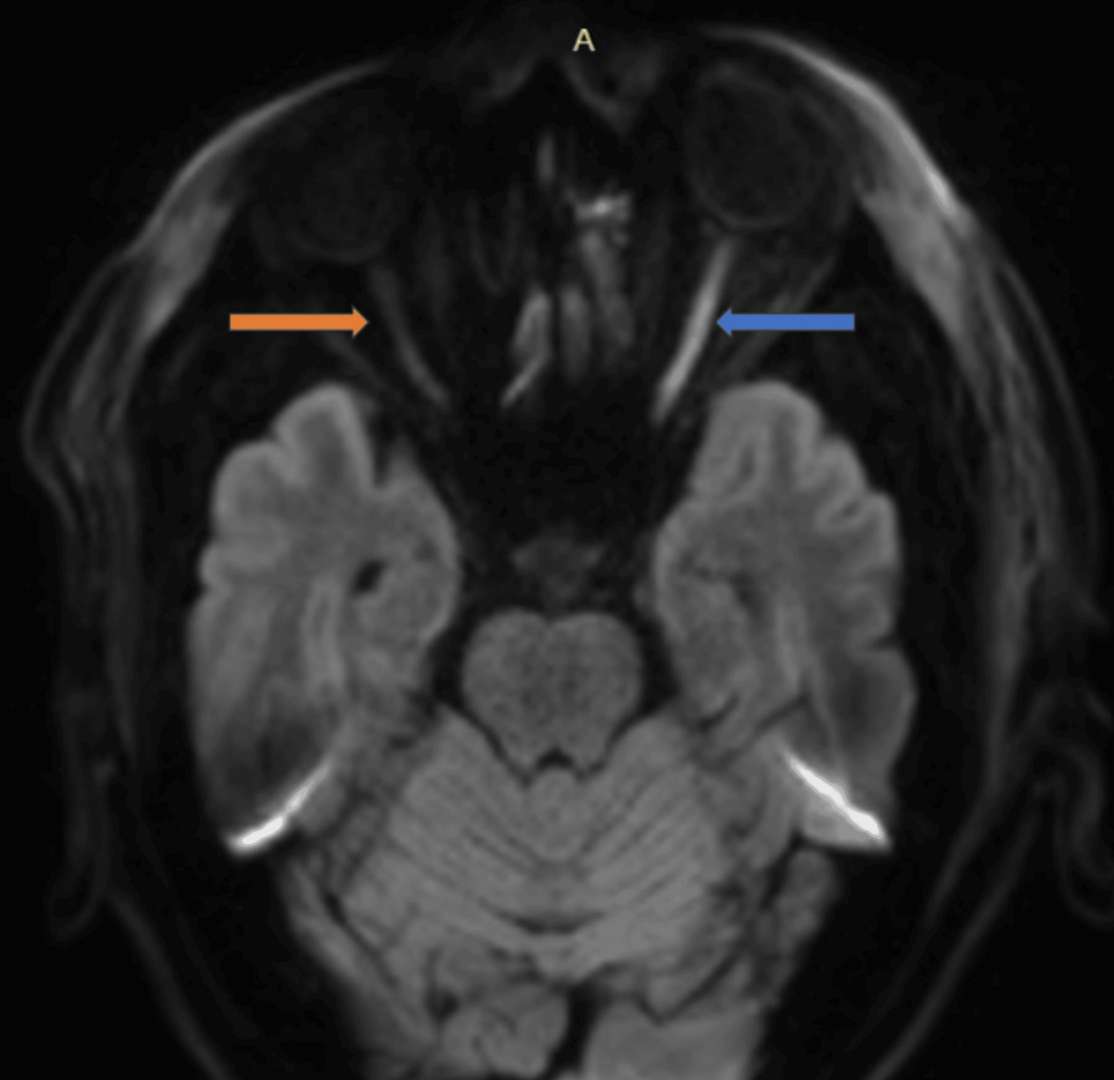
Axial diffusion-weighted image shows the markedly hyperintense signal (diffusion restriction) in the intraorbital segment of the left optic nerve (blue arrow) compared to the normal signal intensity of the right optic nerve (orange arrow).

A 55-year-old male presented with acute vision loss in the left eye following blunt facial trauma. On examination, the patient exhibited no light perception in the left eye, with a significant afferent pupillary defect noted on the same side. There was pronounced swelling, bruising, and tenderness over the left periorbital and maxillary regions. The periorbital hematoma was evident on both clinical evaluation and imaging. The right eye and systemic neurological assessment were unremarkable, and there were no signs of additional cranial nerve involvement or motor and sensory deficits. MRI revealed diffusion restriction in the left optic nerve without any evidence of optic canal fracture or extrinsic compression, as shown in Figure 3.

**Figure 4:**
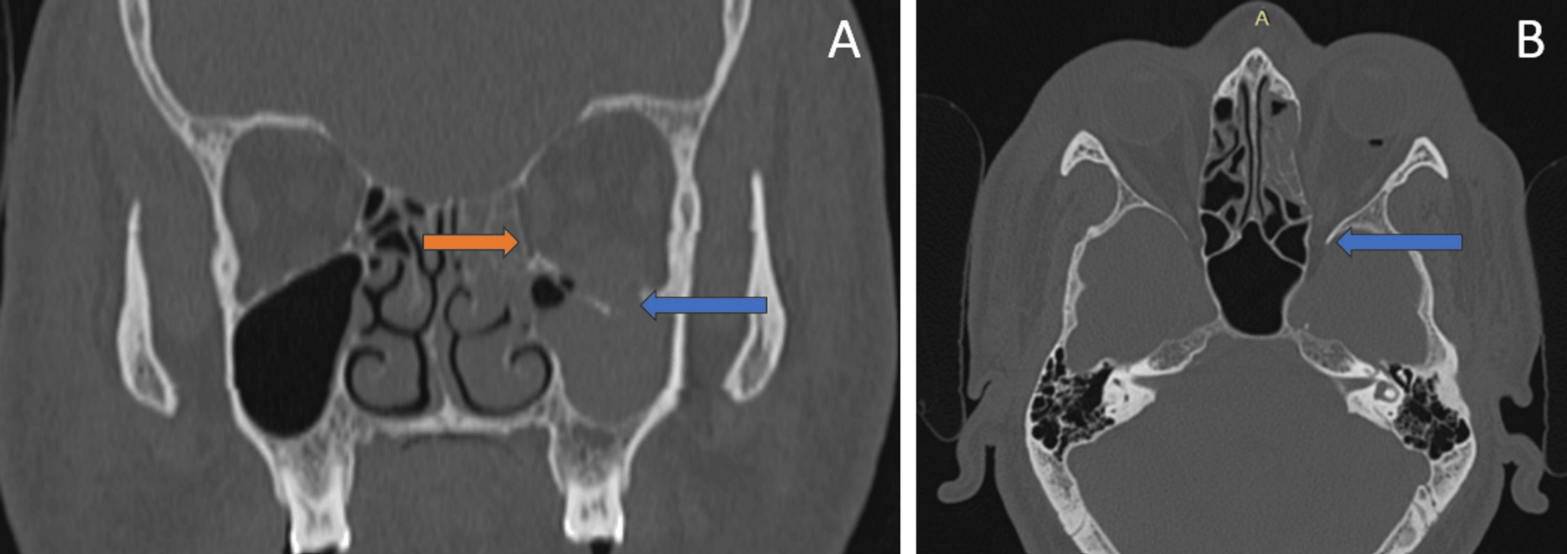
(A) Coronal non-contrast computed tomography image of the orbit shows fracture of the floor (blue arrow) and medial wall (orange arrow) of the left orbit. (B) No fracture of the left optic nerve canal (blue arrow).

CT identified comminuted, mildly displaced fractures of the floor and medial wall of the left orbit, as shown in Figure 4A. Notably, there was no left optic nerve canal fracture, as shown in Figure 4B.

These imaging findings suggested the diagnosis of indirect TON. The patient was treated with a high-dose corticosteroid regimen. Intravenous methylprednisolone was administered at a dose of 1 gram per day, divided into four doses, for five days to address inflammation and prevent secondary ischemic injury to the optic nerve. A tapering course of oral prednisolone followed this at 1 mg/kg per day for three days, gradually reduced over nine days. The therapeutic aim was to stabilize the optic neuropathy and mitigate potential complications associated with trauma-induced optic nerve damage.

During follow-up, the patient demonstrated mild improvement in visual perception in the affected eye, best corrected visual acuity improved to 6/60 on the Snellen visual acuity chart. Repeat imaging showed resolution of retrobulbar hematoma, with no further optic nerve changes or new complications. Despite limited vision recovery, the patient achieved a stable status without progressing symptoms. This outcome highlights the challenges of managing indirect TON and the critical importance of prompt diagnosis and tailored therapy.

Case 3

An 18-year-old male presented with complete vision loss in the left eye following blunt facial trauma. Clinical assessment revealed no light perception in the left eye, accompanied by a significant afferent pupillary defect. The left periorbital region exhibited swelling and tenderness. Systemic neurological examination was unremarkable, with no motor or sensory deficits or involvement of other cranial nerves beyond the ON.

**Figure 5:**
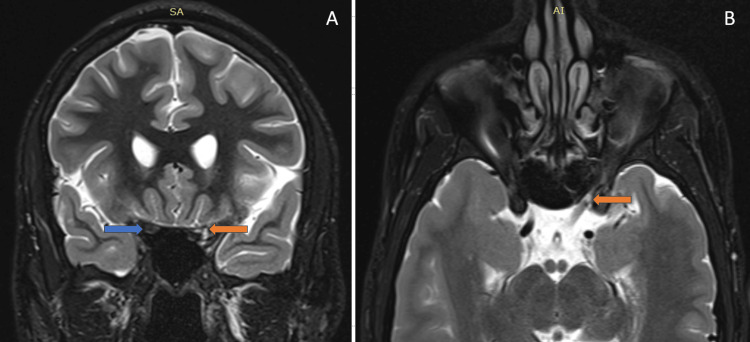
(A) Coronal MRI short tau inversion recovery (STIR) image shows fluid signal intensity in the intracanalicular segment of the left optic nerve suggestive of avulsion/tear of the left optic nerve (orange arrow) compared to the normal signal intensity of the right optic nerve in the optic nerve (blue arrow). (B) Axial MRI STIR image shows fluid signal intensity in the intracanalicular segment of the left optic nerve suggestive of avulsion/tear of the left optic nerve (orange arrow).

MRI of the brain and orbits revealed focal thinning and a suspected discontinuity of the left optic nerve at the junction of the intracanalicular and intracranial segments, consistent with an avulsion/tear, as shown in Figures 5A-5B.

The patient was treated with a high-dose corticosteroid regimen to address inflammation and reduce secondary ischemic injury. Intravenous methylprednisolone was administered at 1 gram/day in four divided doses for five days. This was followed by a transition to oral prednisolone at 1 mg/kg/day for three days, tapered over nine days. Surgical intervention was deferred due to the traumatic avulsion/tear in the optic nerve, as the structural damage was deemed irreparable, and surgery would not have provided any functional benefit. The patient and family were counselled extensively about the guarded prognosis, with an emphasis on the poor likelihood of visual recovery.

Post-treatment follow-up revealed no improvement in visual function in the affected eye. Imaging confirmed the optic nerve's persistent focal thinning and discontinuity at the intracanalicular and intracranial junction. While there was no progression of the optic nerve damage or new complications, the structural injury remained unchanged. The visual prognosis was ultimately poor, consistent with the severity of the traumatic insult and the irreversible nature of the optic nerve damage. This outcome highlights the significant challenges in managing TON with associated nerve tears and underscores the limitations of both medical and surgical interventions in such cases.

Case 4

A 28-year-old male presented with sudden vision loss in the left eye following blunt head trauma. Clinical evaluation revealed no perception of light in the left eye and a significant afferent pupillary defect. Mild periorbital swelling was noted without external globe injury. Neurological examination showed no additional deficits, with intact motor and sensory functions. The patient reported mild discomfort in the orbital region, but the systemic examination was unremarkable.

**Figure 6:**
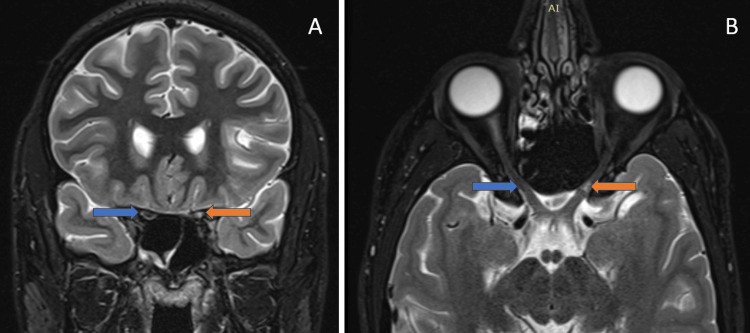
(A) Coronal MRI short tau inversion recovery (STIR) image shows high signal intensity in the intracanalicular segment of the left optic nerve suggestive of traumatic left optic neuropathy (orange arrow) compared to the normal signal intensity of the right optic nerve in the optic nerve (blue arrow). (B) Axial MRI STIR image shows high signal intensity in the intracanalicular segment of the left optic nerve, suggestive of traumatic left optic neuropathy (orange arrow) compared to the normal signal intensity of the right optic nerve in the optic nerve (blue arrow).

MRI of the brain and orbits and CT correlation demonstrated focal thinning and T2/STIR hyperintensity in the intracanalicular segment of the left optic nerve, as shown in Figures 6A-6B. There was no optic canal fracture or extrinsic compression of the optic nerve, consistent with indirect TON.

**Figure 7:**
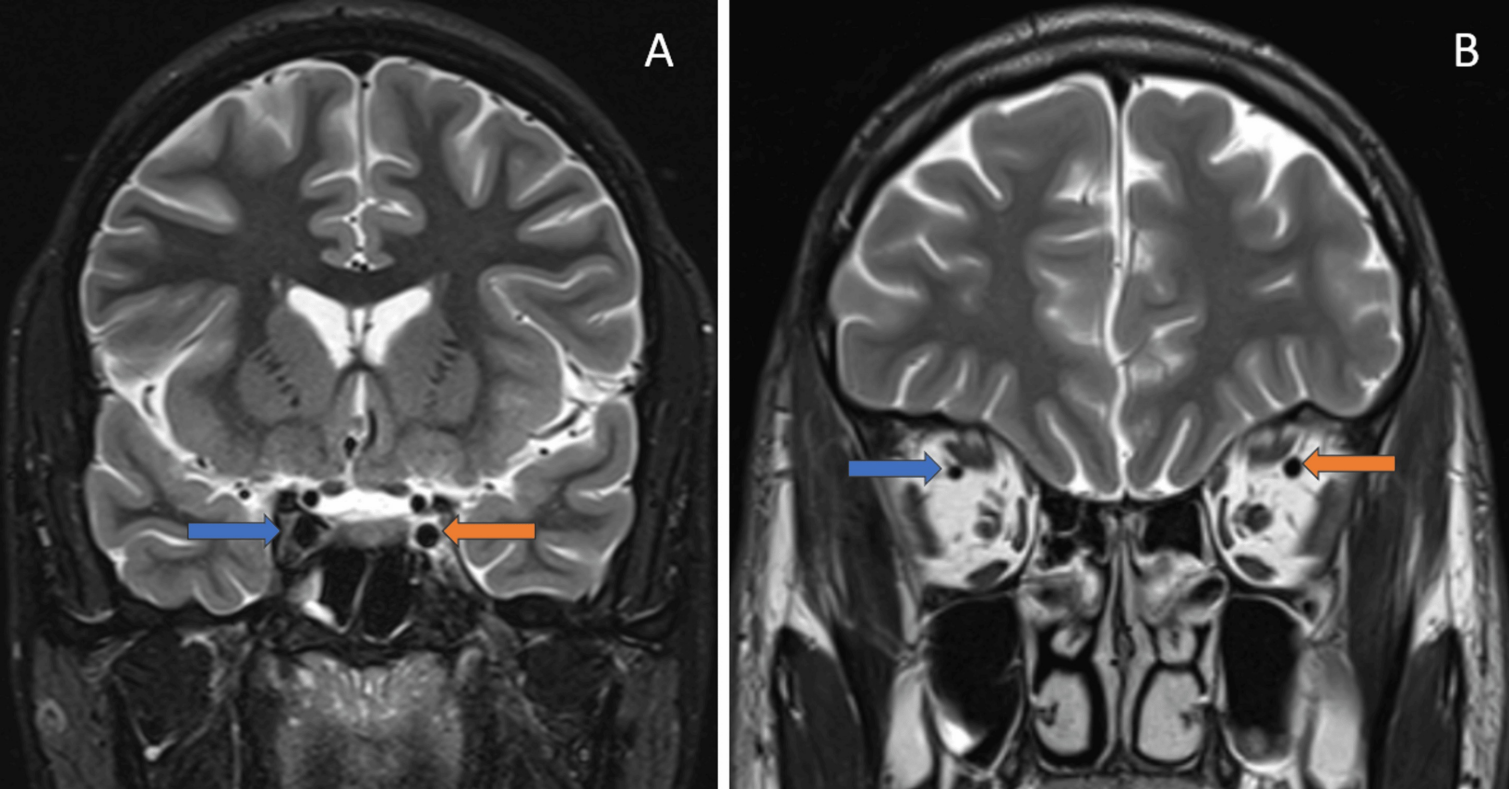
(A) Coronal MRI short tau inversion recovery (STIR) image shows high signal intensity in the left cavernous sinus (orange arrow) compared to the normal signal intensity of the right cavernous sinus (blue arrow). (B) Coronal MRI T2-weighted image shows the prominence of the left superior ophthalmic vein (orange arrow) compared to the normal-sized right superior ophthalmic vein (blue arrow).

The left cavernous sinus exhibited mild STIR hyperintensity with a prominence of the left superior ophthalmic vein, raising suspicion for cavernous sinus thrombosis, as shown in Figures 7A-7B.

The patient received a comprehensive treatment regimen targeting both TON and cavernous sinus thrombosis. Low molecular weight heparin (LMWH) was administered at 100 IU/kg subcutaneously twice daily to address the thrombosis. Intravenous ceftriaxone 1 g was given twice daily to manage potential infectious complications related to sinus involvement. High-dose corticosteroids were employed to mitigate optic nerve inflammation, starting with intravenous methylprednisolone at 1 g/day in four divided doses for five days, followed by a transition to oral prednisolone at 1 mg/kg/day for three days, tapered over nine days. Regular monitoring of visual acuity and neuro-ophthalmological parameters was conducted throughout the treatment course.

Despite aggressive medical management, the patient’s visual function in the left eye did not improve, and there was persistent no perception of light. Follow-up imaging showed resolution of cavernous sinus hyperintensity and normalization of the left superior ophthalmic vein, indicating a resolving thrombosis. However, the thinning and hyperintensity of the left optic nerve persisted, reflecting irreversible structural damage. Craniofacial fractures healed without further complications, and the patient remained stable without additional systemic issues. The poor visual prognosis underscored the severity of the initial optic nerve injury and the limitations of current treatment modalities for TON with cavernous sinus thrombosis.

Case 5

A 30-year-old male presented with bilateral diminished vision, more severe in the right eye, following a traumatic event. Clinical evaluation revealed significant visual impairment with bitemporal hemianopia and an afferent pupillary defect more pronounced on the right. External examination revealed no visible globe trauma and neurological assessment showed no additional deficits.

**Figure 8:**
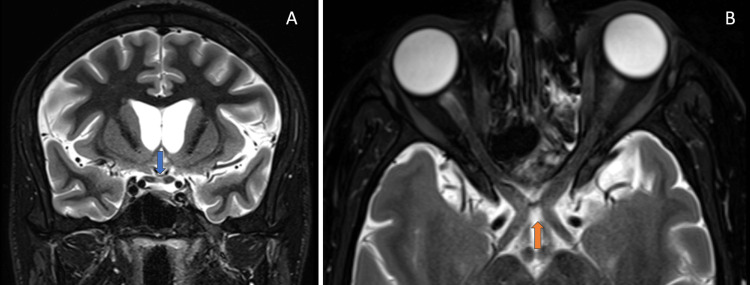
(A) Coronal MRI short tau inversion recovery (STIR) image shows high signal intensity in the optic chiasm (blue arrow). (B) Axial MRI STIR image shows high signal intensity in the optic chiasm (orange arrow).

MRI of the orbits shows focal thinning and STIR hyperintensity involving the decussating fibres of the optic chiasm, consistent with traumatic chiasmal syndrome, as shown in Figures 8A-8B.

The patient was treated with a high-dose corticosteroid regimen to reduce inflammation and mitigate secondary ischemic damage to the optic chiasm and nerves. Intravenous methylprednisolone was administered at a dose of 1 gram/day in four divided doses for five days. This was followed by a transition to oral prednisolone at 1 mg/kg/day for three days, tapered over nine days. A multidisciplinary team involving ophthalmology, neurology, and neurosurgery was involved in the patient’s care, with a plan for follow-up imaging and functional assessments.

Despite early intervention, the patient experienced only minimal improvement in visual function, visual acuity improved to 3 meters finger counting on the left side and light perception in the right eye. Follow-up imaging demonstrated persistent thinning and hyperintensity of the optic chiasm, indicating irreversible structural damage. While the patient stabilized neurologically, the prognosis for substantial visual recovery remained poor due to the severity of the chiasmal injury and associated craniofacial trauma. This outcome underscores the challenges in managing traumatic chiasmal syndrome and the limited efficacy of current therapeutic strategies in cases with severe structural damage.

## Discussion

TON encompasses a spectrum of optic nerve injuries following craniofacial trauma, varying in severity, mechanism, and prognosis. This case series highlights five distinct presentations: direct TON, indirect TON, optic nerve tear, TON with cavernous sinus thrombosis, and traumatic chiasmal syndrome, offering a window into the diagnostic and therapeutic challenges associated with these injuries.

TON can result from direct or indirect trauma. Direct TON involves anatomical disruption of the optic nerve due to penetrating injuries or bone fragments from fractures, as seen in Case 1. Indirect TON, the most common variant, results from transmitted forces causing shearing at the junctions of mobile and fixed optic nerve segments, often involving the intracanalicular portion (71.4% of cases), as seen in Cases 2 and 4 [2,3]. Optic nerve tears, like in Case 3, represent the extreme end of direct injury, characterized by discontinuity of nerve fibres due to avulsion. Cavernous sinus thrombosis associated with TON, as in Case 4, reflects the interplay of trauma-induced thrombosis and inflammatory sequelae, compounding the optic nerve damage.

Traumatic chiasmal syndrome (Case 5) is a rare variant in which severe impact damages the crossing fibres of the chiasm, typically resulting in bitemporal hemianopia [6,7]. Such injuries frequently accompany skull base fractures, including anterior cranial fossa involvement, and may be associated with cerebrospinal fluid (CSF) leaks.

High-resolution imaging plays a pivotal role in TON diagnosis and management. CT remains the gold standard for evaluating craniofacial trauma and associated bone injuries. High-resolution thin-section axial CT with multiplanar reformation enables visualization of optic canal fractures, orbital wall disruptions, and bone fragment displacement [8,9], essential for surgical planning in cases like direct TON (Case 1). MRI, with STIR and diffusion-weighted sequences, is superior in detecting optic nerve oedema, thinning, or tears, as demonstrated in Cases 2, 3 and 4 [10]. For cavernous sinus thrombosis (Case 4), T2-weighted and STIR sequences demonstrated venous congestion and superior ophthalmic vein dilation. MRI confirms chiasmal thinning and hyperintensities in traumatic chiasmal syndrome, as seen in Case 5, correlating with visual field defects such as bitemporal hemianopia [11].

Emerging imaging techniques, including diffusion tensor imaging (DTI) and optical coherence tomography (OCT), hold the potential for detecting early axonal damage and tracking progression [12]. OCT is particularly useful in chronic cases to document retinal nerve fibre layer (RNFL) thinning​ [13]. DTI may quantify microstructural changes in the optic nerve, providing prognostic insights [14].

Management of TON and related syndromes is complex and multifaceted, requiring a combination of medical, surgical, and supportive therapies tailored to the specific mechanism and severity of injury [15]. Corticosteroids remain the most commonly used medical intervention for TON, with high-dose methylprednisolone employed to reduce inflammation, oedema, and oxidative stress. In this case series, intravenous methylprednisolone (1 g/day in four divided doses for five days) followed by oral prednisolone (1 mg/kg/day for three days, tapered over nine days) was administered across all cases. Despite their widespread use, the efficacy of corticosteroids remains controversial. While they appeared beneficial in reducing inflammation in cases of indirect TON and traumatic chiasmal syndrome, their impact was limited in cases with severe structural damage, such as optic nerve tears. This aligns with findings from studies like the International Optic Nerve Trauma Study, which have not demonstrated clear superiority of corticosteroids over observation, particularly in indirect injuries or those involving profound structural disruption [16]. Moreover, corticosteroid use must be carefully weighed against potential risks, including gastrointestinal bleeding, infections, and complications in patients with traumatic brain injuries [16].

Surgical decompression is an important intervention in cases where imaging confirms optic nerve compression, such as bony impingement or optic canal narrowing [17]. In this series, EOND was performed in a patient with direct TON, leading to visual improvement. The timing of surgery is critical, with early intervention (<24-48 hours) offering the best outcomes in patients with residual light perception [16]. However, surgery was deferred in cases of optic nerve tear and traumatic chiasmal syndrome, as the structural damage was considered irreparable and surgical intervention unlikely to provide functional benefits. For TON associated with cavernous sinus thrombosis, such as in Case 4, anticoagulation with LMWH (100 IU/kg twice daily) was essential to resolve the thrombosis and prevent further complications. This was paired with intravenous ceftriaxone to address potential septic complications arising from sinus involvement. Such multimodal therapy highlights the need for individualized management strategies based on the underlying pathology and associated risks.

The outcomes in this series reflect the variability in prognosis for TON and related syndromes. Patients with indirect TON and those undergoing timely surgical decompression for direct TON showed some degree of visual recovery, while cases involving severe structural damage or vascular complications, such as optic nerve tears or cavernous sinus thrombosis, had poor visual outcomes. These findings are consistent with existing literature, emphasising that factors such as initial visual acuity, time to intervention, and the presence of optic canal fractures are key determinants of prognosis [15,16]. Emerging therapies, including neuroprotective agents like erythropoietin and brain-derived neurotrophic factor (BDNF), as well as experimental approaches like stem cell therapy, hold promise for addressing the limitations of current treatment modalities [18]. Such therapies aim to mitigate secondary axonal degeneration and promote optic nerve repair, offering hope for improved outcomes in severe cases.

## Conclusions

This case series highlights the diverse presentations and management challenges of TON and its related syndromes. In the case of direct TON associated with optic canal fracture and bony fragment compressing the optic nerve, surgical decompression combined with corticosteroid therapy resulted in partial visual recovery, emphasizing the importance of timely intervention when mechanical compression is present. Indirect TON without optic canal fracture showed stabilization but limited recovery, reflecting the difficulty in managing ischemic damage in non-compressive injuries. Optic nerve tears, as seen in one case, underscored the irreversible nature of severe structural damage, where surgical intervention was not viable, and vision restoration was not achieved. The case of TON with cavernous sinus thrombosis demonstrated thrombosis resolution with anticoagulation and antibiotics but no visual improvement, highlighting the complexity of managing vascular and inflammatory components simultaneously. Finally, traumatic chiasmal syndrome, presenting with bitemporal hemianopia, showed minimal response to corticosteroid therapy, with imaging confirming persistent chiasmal damage.

The findings underscore the critical role of CT and MRI in diagnosing the extent and mechanism of optic nerve injuries, which guide management strategies. While corticosteroids and surgical interventions remain foundational treatments, the prognosis depends heavily on the severity of initial injury and structural damage. This series highlights the need for early, accurate diagnosis and the limitations of current therapeutic options in cases of severe nerve disruption.
